# An assessment of auditory-guided locomotion in an obstacle circumvention task

**DOI:** 10.1007/s00221-016-4567-y

**Published:** 2016-02-15

**Authors:** Andrew J. Kolarik, Amy C. Scarfe, Brian C. J. Moore, Shahina Pardhan

**Affiliations:** Centre for the Study of the Senses, Institute of Philosophy, University of London, Senate House, Malet Street, London, WC1E 7HU UK; Vision and Eye Research Unit (VERU), Postgraduate Medical Institute, Anglia Ruskin University, Eastings 204, East Road, Cambridge, CB1 1PT UK; Department of Psychology, University of Cambridge, Downing Street, Cambridge, CB2 3EB UK; Department of Clinical Engineering, Medical Imaging and Medical Physics Directorate, Sheffield Teaching Hospitals NHS Foundation Trust, Sheffield, UK

**Keywords:** Obstacle avoidance, Navigation, Echolocation, Locomotion, Central nervous system

## Abstract

This study investigated how effectively audition can be used to guide navigation around an obstacle. Ten blindfolded normally sighted participants navigated around a 0.6 × 2 m obstacle while producing self-generated mouth click sounds. Objective movement performance was measured using a Vicon motion capture system. Performance with full vision without generating sound was used as a baseline for comparison. The obstacle’s location was varied randomly from trial to trial: it was either straight ahead or 25 cm to the left or right relative to the participant. Although audition provided sufficient information to detect the obstacle and guide participants around it without collision in the majority of trials, buffer space (clearance between the shoulder and obstacle), overall movement times, and number of velocity corrections were significantly (*p* < 0.05) greater with auditory guidance than visual guidance. Collisions sometime occurred under auditory guidance, suggesting that audition did not always provide an accurate estimate of the space between the participant and obstacle. Unlike visual guidance, participants did not always walk around the side that afforded the most space during auditory guidance. Mean buffer space was 1.8 times higher under auditory than under visual guidance. Results suggest that sound can be used to generate buffer space when vision is unavailable, allowing navigation around an obstacle without collision in the majority of trials.

## Introduction

Vision provides important information for locomotion, allowing individuals to detect and walk around obstacles in the path of travel, and avoid collisions. Although vision loss can cause substantial reduction in spatial awareness, audition is capable of offering a rich source of spatial information when vision is degraded or lost (Zahorik et al. 2005). Relatively little is known regarding how sound informs dynamic obstacle clearance and how accurately sound can inform the locomotor system in order to avoid collisions (Kolarik et al. 2014a). This study investigated whether sound could be used in the absence of vision to circumvent an obstacle obstructing the path of travel, a task frequently encountered during daily locomotion (Gérin-Lajoie et al. 2008; Hackney et al. 2014).

Some blind humans echolocate by emitting sounds and use the returning echoes to obtain spatial information (Rosenblum 2011; Teng et al. 2012), and this skill can be used for navigation. Wallmeier and Wiegrebe (2014b) showed that during echolocation sighted and blind participants orient the body and head towards a desired location. Fiehler et al. (2015) showed that blind echolocation experts performed better than blind and sighted novices in a task involving listening to pre-recorded binaural echolocation clicks that were created while walking along a corridor, and reporting the direction of the corridor (left, right, or straight ahead). Strelow and Brabyn (1982) reported that blind and normally sighted blindfolded participants could use sounds to travel parallel to a wall. Performance was poorer when a line of poles was used rather than a wall. Gordon and Rosenblum (2004) showed that normally sighted blindfolded participants were able to use acoustic information to judge whether apertures of various width could afford passage without turning their body. Kolarik et al. (2014b) investigated navigation using echoic sensory substitution devices (SSDs), which are electronic travel aids based on an echolocation principle that locate silent objects (these are distinct from “visual pattern” SSDs, that convert visual information into an auditory signal using a predetermined transformation algorithm). They reported that echoic information informed the degree of shoulder rotation required by blindfolded sighted participants to move through narrow apertures. When near a wall, low-frequency sound informed locomotion by blind individuals to allow them to walk parallel to the wall (Ashmead et al. 1998). Ashmead et al. (1989) examined how congenitally blind children walked along a path that was sometimes obstructed by an obstacle. Children spent longer in front of the obstacle than behind, suggesting they perceived the presence of the obstacle, and spent more time in front in order to notice and navigate around it. However, they were not given specific instructions regarding whether they should make sounds or what they should do regarding the obstacle, and they contacted it in approximately half of the trials.

Several studies have investigated whether audition informs locomotion for blind (Supa et al. 1944; Worchel et al. 1950; Worchel and Mauney 1951; Ammons et al. 1953) and blindfolded sighted participants (Supa et al. 1944; Carlson-Smith and Weiner 1996; Rosenblum et al. 2000) using an obstacle approach and detection task. Although collisions did sometimes occur, participants were able to use sound to approach a large obstacle such as a wall, report when they first perceived it, approach it as close as possible without touching it, and to stop in front of it to face it. Various sounds could be used to perform the task, including shoe clicks on a hardwood floor (Supa et al. 1944; Worchel and Dallenbach 1947) or linoleum (Carlson-Smith and Weiner 1996), sounds from shoes when walking along a concrete path (Worchel et al. 1950; Worchel and Mauney 1951; Ammons et al. 1953), or a sound chosen by the participant, such as mouth clicks or the words “hello” or “check” (Rosenblum et al. 2000). Detection was also possible with sounds from feet wearing stockings on a carpet, although performance was poorer than that with shoes on a hardwood floor (Supa et al. 1944).

In real-life travel situations, individuals often have to detect and then circumvent an obstacle. An assessment of the ability to use sound for this is the focus of the present study. Obstacle circumvention is arguably a more difficult task than obstacle approach and detection, since the former requires the individual to detect the obstacle’s position and edges, perform precise motor actions to move around it, and allow adequate space between the obstacle and the body at the point of moving past it to ensure safe navigation. Research in the visual domain has indicated that individuals maintain a protective envelope around themselves when moving around obstacles (Gérin-Lajoie et al. 2008; Hackney et al. 2014), constituting the personal space around the body when walking (Gérin-Lajoie et al. 2005). In addition, full vision allows passing on the side of the obstacle affording most space (Fajen and Warren 2003; Hackney et al. 2014). The term “buffer space”, used by Franchak et al. (2012) to describe the margin between the body and sides of an aperture during locomotion, is used here to denote the space between the shoulders and the obstacle. It is currently unknown whether sound allows participants to generate a buffer space and to pass on the side affording the most space.

Locomotion under auditory guidance is often relatively slow (Strelow and Brabyn 1982). However, movement indices including buffer space, movement time, and number of velocity corrections have not previously been assessed for obstacle approach and circumvention using sound. In the current study, a Vicon motion capture system was utilized to formally quantify these indices to enable greater insight regarding how useful auditory-guided locomotion is for real-life travel situations, for example by quantifying how large a margin of safety is needed when avoiding an obstacle using sound, and to provide a performance baseline for rehabilitation training for auditory-guided locomotion following visual loss.

In summary, the aim of the current study was to build on and extend previous work investigating auditory-guided locomotion in indoor environments (Supa et al. 1944; Strelow and Brabyn 1982; Carlson-Smith and Weiner 1996) by assessing the obstacle circumvention performance of blindfolded sighted echo-naïve participants generating mouth click sounds. Visually guided locomotion was used as a baseline. We hypothesized that sound would provide spatial information allowing participants to circumvent an obstacle in their path accurately and safely. Compared to visual guidance, auditory guidance was predicted to result in greater space between the shoulders and the obstacle (buffer space), more velocity corrections, and longer movement times reflecting a more cautious approach, as well as an increased number of impacts. An additional “no-click” control experiment was conducted, to compare obstacle circumvention using mouth clicks to that using ambient sound or footfalls on the carpet.

## Methods

### Participants

There were 10 participants in the main experiment (seven males and three females, mean age 31 years, range 21–42 years). For the “no-click” control experiment, there were nine participants (seven males and two females, mean age 34 years, range 26–42 years, six of whom took part in the main experiment). All reported normal or corrected to normal vision, and had normal hearing, defined as better-ear average (BEA) hearing threshold across the frequencies 500, 1000, 2000 and 4000 Hz ≤ 15 dB HL, as measured using an Interacoustics AS608 audiometer following the procedure recommended by the British Society of Audiology (2011). None of the participants reported any prior experience using self-generated sounds to perceive objects. The experiments followed the tenets of the Declaration of Helsinki. Written informed consent was obtained from all individual participants, following an explanation of the nature and possible consequences of the study. The experiments were approved by the Anglia Ruskin University Ethics committee.

### Apparatus and data acquisition

Participants were tested in a quiet room measuring 5.7 × 3.5 m with a ceiling height of 2.8 m, and with an ambient sound level of approximately 36 dBA. The floor was carpeted, the walls were painted, and the ceiling was tiled. Obstacles were flat, rectangular, movable, and constructed of wood covered by smooth aluminium foil, following Arnott et al. (2013). The aluminium foil provided high reflectivity, but also produced near-specular reflection of sounds, perhaps making the obstacle more difficult to localize than bare wood. A small practice obstacle was used for training (width 0.5 × height 0.34 m), and a large obstacle was used for both training and testing (width 0.6 × height 2 m). The obstacles were located close to the centre of the room. Silence was maintained during testing, except when participants produced self-generated mouth clicks.

Three-dimensional kinematic data were collected at 50 Hz using an 8-camera motion capture system (Vicon Bonita; Oxford Metrics Ltd). Retro-reflective spherical markers were attached to the participant, bilaterally, at the following anatomical locations: the most distal, superior aspect of the first toe, the most distal, superior aspect of the fifth toe, the posterior aspect of the calcanei, the acromioclavicular joint, and antero-lateral and postero-lateral aspects of the head. Single markers were placed on the sternum and the posterior aspect of the dominant hand. Three additional markers were attached to the front aspect of the obstacle to determine the width and location of the obstacle within the laboratory coordinate system; these remained attached throughout the experiment. Marker trajectory data were filtered using the cross-validatory quintic spline smoothing routine, with “smoothing” options set at a predicted mean squared error value of 10 and processed using the Plug-in gait software (Oxford Metrics Ltd). Due to the aluminium foil covering the obstacle, incorrect reflections were sometimes recorded by the Vicon system. However, individual markers were carefully labelled for each trial to exclude invalid reflections from the analyses.

Key variables relating to obstacle circumvention were assessed either using custom-written Visual Basic scripts or recorded by the experimenter. Definitions are presented in Table 1.

**Table 1:** Assessed dependent variables and their descriptions

Variable	
*Obstacle present trials*	
Buffer space	Medio-lateral distance between the shoulder marker and obstacle at the point of crossing the obstacle (defined as the point where the shoulder marker passes the marker attached to the front aspect of the obstacle)
Movement time	Time taken to complete movement measured from when the sternum marker was 1 m away from the obstacle in the anterior-posterior direction until the point of crossing the obstacle marker
Velocity corrections	Number of changes in forward velocity from when the sternum marker was 1 m away from the obstacle until the point of crossing the obstacle marker
Obstacle detections	The experimenter recorded trials where the participant raised their hand marker to indicate obstacle perception
Obstacle detection range	Anterior-posterior distance between the participant’s sternum marker and obstacle marker, measured at the point at which the participant raised their hand marker to indicate obstacle perception
Collisions	The experimenter recorded trials where a collision occurred between any part of the participant’s body and the obstacle
Side of obstacle avoidance	The side of avoidance of the obstacle by the participant was recorded by the experimenter
*Obstacle absent trials*	
False perceptions	Number of obstacle absent trials in which the participant raised their hand marker to falsely indicate obstacle perception

### Procedures

Participants were instructed that they would first take part in an auditory guidance condition (which included a training session) that involved using sound to perceive and navigate around an obstacle, followed by a visual guidance condition. Following Kolarik et al. (2014b), the visual condition was presented last to avoid the participants becoming familiar with the range of obstacle positions and distances prior to the auditory guidance condition.

The auditory guidance condition began with static and dynamic training in order for the participants to become familiar with producing echolocation clicks and listening to them when navigating. During static training, participants were seated in the centre of the room with the small practice obstacle placed on a table in front of them at head height at a distance of 25 cm. The obstacle was randomly placed in front of them or was absent. Participants practised generating mouth clicks and using the sound echoes to detect the presence or absence of the practice obstacle. Although alternative sounds such as finger snaps, hand claps, or mechanical sounds such as those produced by tapping a cane against the floor can be used for echolocation, mouth clicks were chosen in the current study following Rojas et al. (2009), who hypothesized that mouth clicks offer several advantages to alternative sounds, providing relatively accurate distance estimation and usability. Also, mouth clicks can be generated at high sustained repetition rates. The static training lasted for a minimum of 15 min; for the first 5 min, the participants were allowed to keep their eyes open, for the following 5 min they were encouraged to close their eyes, and for the last 5 min they were blindfolded (Kolarik et al. 2014b).

The dynamic training phase consisted of practising navigating around the large obstacle using mouth clicks from an approach distance of 1.75 m. Participants trained for a minimum of 15 min, split into three sessions (1. full vision, 2. eyes closed, 3. blindfolded) lasting 5 min each. The centre of the obstacle was located on the midline relative to the participant (straight ahead) for the first 10 min of training, and varied randomly in location relative to the participant, at the midline, or 25 cm to the right or left during the last 5 min of training when the participant was blindfolded.

Obstacle detection performance was assessed prior to testing with locomotion. Participants were positioned facing the location where the obstacle might be and at 25 cm from that location, and were asked to produce mouth clicks and to report whether the obstacle was present. Twenty trials were performed. In half of the trials (randomized), the large obstacle was present and in the other half it was absent. Mean performance was 90 % (SD = 11 %). All participants scored 70 % or more, and four participants scored 100 %.

**Fig. 1:**
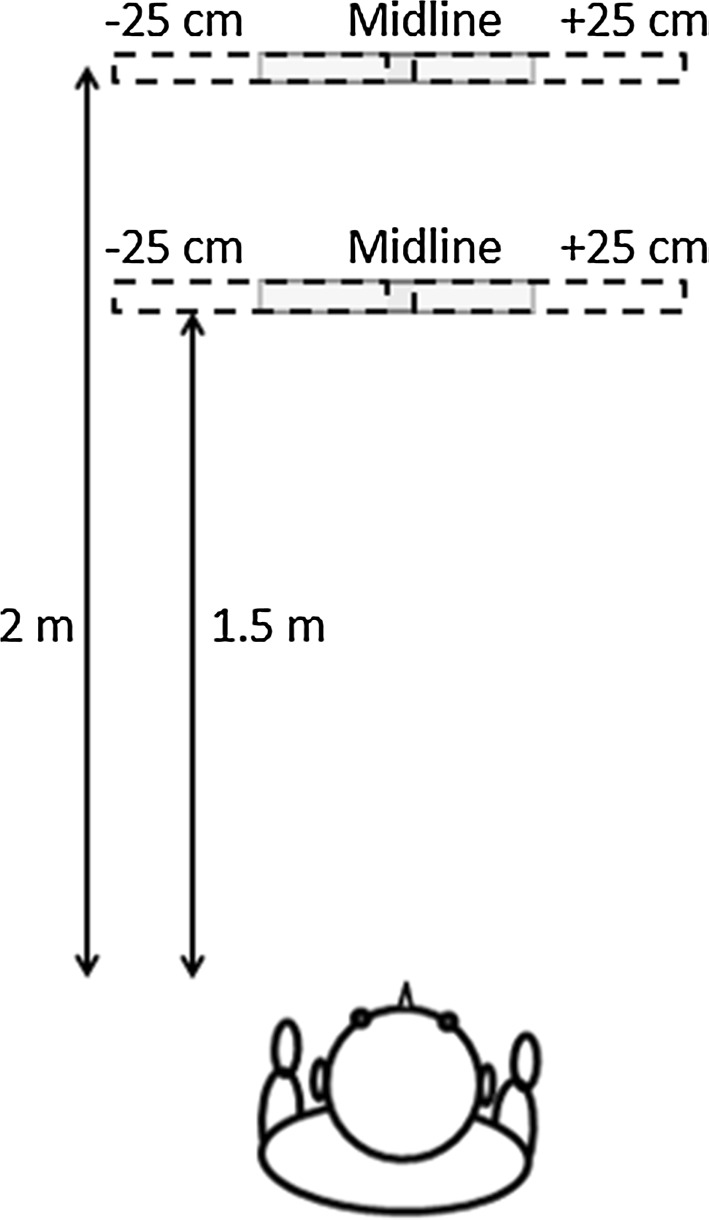
Schematic diagram of the experimental setup. The obstacle was placed either on the midline or 25 cm to the *left* or *right* relative to the participant. The approach distance was either 1.5 or 2 m. The obstacle was 0.6 m wide

The large obstacle was used in the main testing phase. The layout is illustrated in Fig. 1. Participants were instructed that they would be blindfolded, would need to walk forward while generating clicks, and should report if an obstacle was present in their path. They were asked to maintain a straight line of travel until the obstacle was first perceived, report when they first perceived the obstacle by raising their dominant hand (similar to the obstacle approach and detection task used by Supa et al. 1944; Ammons et al. 1953), and then to circumnavigate the obstacle without collision. They were informed that the obstacle would sometimes be absent. Trials were terminated when the participant moved past the obstacle or if a collision occurred. The position of the large obstacle was chosen randomly from three possibilities: on the midline, or 25 cm to the right or left. To avoid the participants anticipating the distance to the obstacle, the distance was randomly set to 1.5 or 2 m (Fig. 1). Each participant performed three trials for each obstacle location. In total each participant completed 24 trials, including six no-obstacle “check” or “false” trials (Worchel et al. 1950; Worchel and Mauney 1951; Ashmead et al. 1989). Use of the hands to touch the obstacle was not allowed. Participants’ ears were occluded between trials using ear-defender constructed headphones. The start of the trial was signalled by a shoulder tap from the experimenter. At the start of each trial, a removable plastic box was used so that participants aligned their feet facing forward. Participants were taken back to the starting position by the experimenter, who stood in the same place during each trial, against the wall to the right of the starting point.

When the auditory guidance trials had been completed, participants took part in a full- vision condition, similar to that used by Hackney et al. (2014). This was identical to the main testing phase of the auditory condition, except that participants did not produce mouth clicks and wore blindfolds only between trials. No feedback was provided during the testing phase, and data were obtained in a single session lasting approximately 1.5 h.

The “no-click” control experiment was identical to the testing phase of the auditory guidance condition of the main experiment, except that participants did not produce mouth clicks, and completed 7 trials (one for each obstacle position and 1 with the obstacle absent), obtained in a single session lasting approximately 20 min.

### Statistical analysis

Unless otherwise specified, data were analysed using repeated-measures ANOVAs to investigate how buffer space, side (right or left) of obstacle avoidance, movement time, and number of velocity corrections were affected by guidance condition (audition and vision), obstacle lateral location (left, midline, or right relative to the participant), and repetition (1–3). The level of significance was chosen as *p* < 0.05. A preliminary analysis showed that scores for all measures were not significantly different for the two approach distances (*p* > 0.05), so the results for these were pooled. Proportional data for side of avoidance were subjected to arcsine transformation, as recommended by Howell (1997). Post hoc analyses were performed using Bonferroni correction.

## Results

Overall, under auditory guidance for trials where the obstacle was present, participants detected the obstacle on 85 % (SD = 11 %) of trials and circumvented the obstacle without collision on 67 % (SD = 21 %) of trials. Participants made no collisions under visual guidance.

**Fig. 2:**
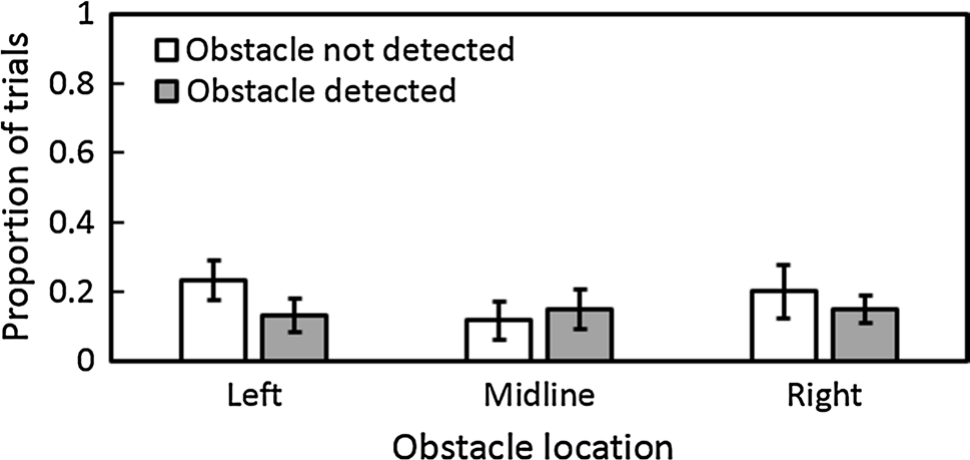
Proportion of obstacle-present trials for which collisions occurred for each obstacle location under auditory guidance. *Open* and *grey* *bars* show the proportions of trials where the obstacle was either not detected or detected prior to collision, respectively. In this and subsequent figures, *error bars* represent ±1 SE

Figure 2 shows the proportion of obstacle-present trials where collisions occurred under auditory guidance. For trials where a collision occurred, the proportion of trials on which the obstacle was detected did not differ significantly from the proportion of trials on which it was not [χ^2^ (2) = 1.51, ns]. Two trials under auditory guidance were discarded from the analyses since the participant did not begin the trial facing directly forwards for these two. The percentage of false reports of obstacle presence in the auditory condition was relatively small at 10 % (SD = 18 %). For the “no-click” control experiment, success was relatively low compared to that with mouth clicks; for obstacle-present trials, participants detected the obstacle on 33, 28, and 17 % of trials for obstacles positioned to the left, midline, and right, respectively, and circumvented the obstacle without collision on 22, 22, and 6 % of trials, respectively. Participants falsely perceived the obstacle to be present on 6 % of obstacle-absent trials. With mouth clicks, participants detected the obstacle on 87, 85, and 85 % of trials, respectively, and circumvented the obstacle without collision on 63, 73, and 65 % of trials, respectively. These results suggest that participants mainly relied on information from mouth click sounds to perform the task in the main experiment.

**Fig. 3:**
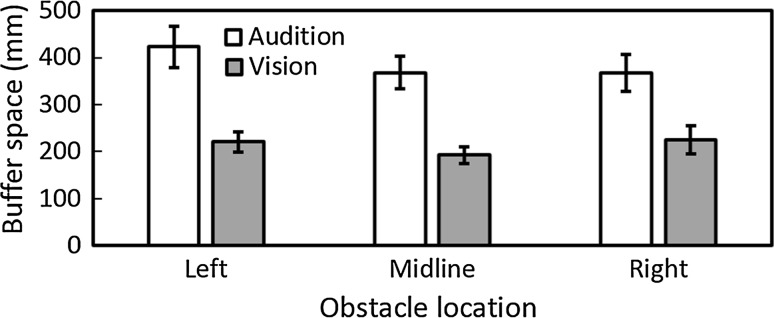
Mean buffer space at the time of crossing under auditory (*open bars*) and visual (*grey bars*) guidance for each obstacle location

**Fig. 4:**
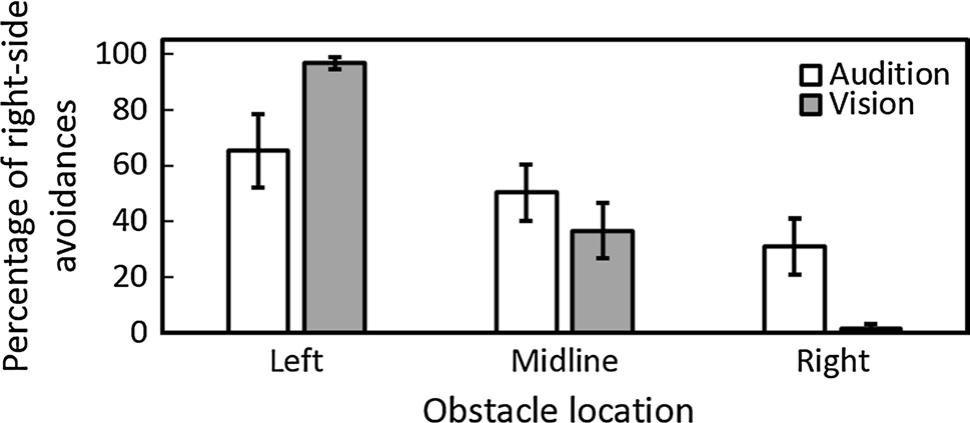
Percentage of right-side avoidances under auditory (*open bars*) and visual (*grey bars*) guidance for each obstacle location

For obstacle-present trials where collisions did not occur, the mean buffer space at the point of crossing the obstacle was larger with auditory guidance than for vision for all obstacle locations (Fig. 3). Under visual guidance, the mean buffer space was 22, 19, and 23 cm for obstacles located to the left, midline, and right, respectively, while it was 42, 37, and 37 cm, respectively, for auditory guidance. There was a significant main effect of guidance condition [*F*(1, 9) = 48.28, *p* = 0.001] and an interaction between guidance condition and repetition [*F*(2, 18) = 5.51, *p* = 0.014]. However, with Bonferroni correction, post hoc comparisons indicated that there were no significant differences in buffer space in either guidance condition across trials. The percentage of right-side avoidances was 97, 37, and 2 % for obstacles located to the left, midline, or right, respectively, under visual guidance, while it was 65, 50, and 31 %, respectively, under auditory guidance (Fig. 4). These results show that participants did not always pass the obstacle on the side affording most space under auditory guidance. Under visual guidance, participants almost always moved to the side of the obstacle that afforded the most space. An analysis of side of avoidance showed a main effect of obstacle location only [*F*(2, 18) = 21.67, *p* = 0.001] and a significant interaction between obstacle location and guidance condition [*F*(2,18) = 8.48, *p* = 0.003]. As expected, in the vision condition participants chose to circumvent the obstacle towards the right side significantly more often when the obstacle was on the left or on the midline than when it was on the right, and when the obstacle was on the midline compared to the right (*p* < 0.001). However, these differences were not significant for the auditory condition.

**Fig. 5:**
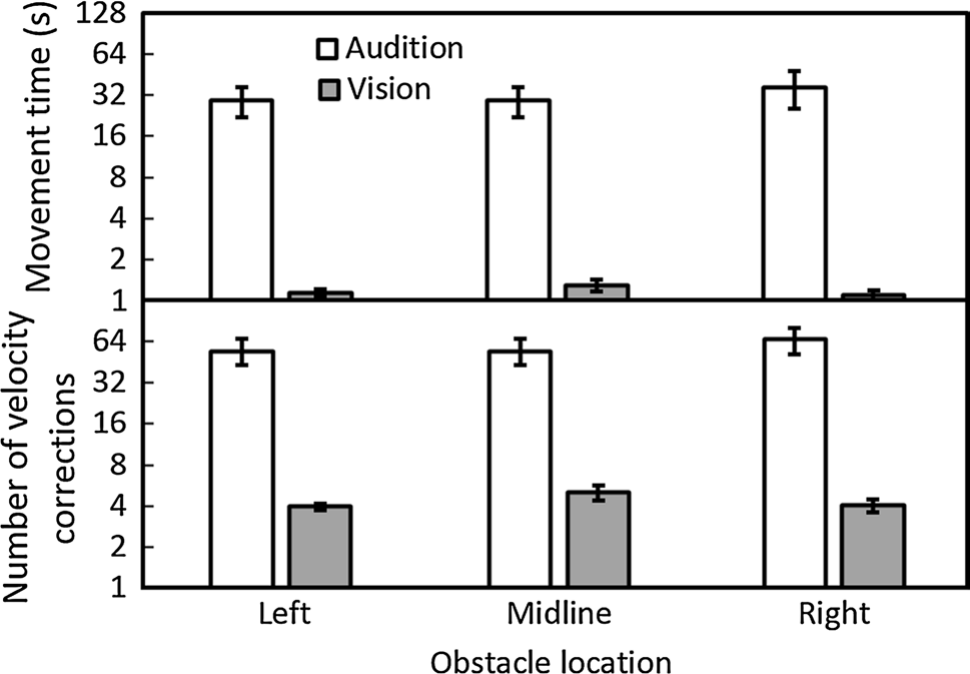
Mean movement time to pass the obstacle (*upper panel*) and mean number of velocity corrections (*lower panel*) under auditory (*open bars*) or visual (*grey bars*) guidance for each obstacle location. The *y* axis is plotted on logarithmic coordinates

As would be expected, both the mean movement times to pass the obstacle (Fig. 5, upper panel) and the mean number of velocity corrections (Fig. 5, lower panel) were considerably greater under auditory guidance than under visual guidance. Under auditory guidance, the mean movement times were 30, 29, and 37 s for obstacles located to the left, midline, and right, respectively, while under visual guidance they were 1.1, 1.3, and 1.1 s, respectively. There was a main effect of guidance condition only [*F*(1, 9) = 14.75, *p* = 0.004]. Under auditory guidance, the mean numbers of velocity corrections were 55, 55, and 66 for obstacles located to the left, midline, and right, respectively, while under visual guidance they were 4, 5, and 4, respectively. There was a main effect of guidance condition only [*F*(1, 9) = 22.20, *p* = 0.001]. Participants raised their hand under auditory guidance to indicate they had perceived the obstacle, as they had to avoid generating sounds other than mouth clicks. This may have resulted in them slowing down in some trials, leading to a small increase in movement time and an increase in one or two velocity corrections compared to visual guidance, where no hand raise was required. However, this would not be sufficient to account for the substantial differences in movement time and velocity corrections between visual and auditory guidance. Increased movement times and velocity corrections confirm that participants took a more cautious approach under auditory guidance than when using vision.

**Fig. 6:**
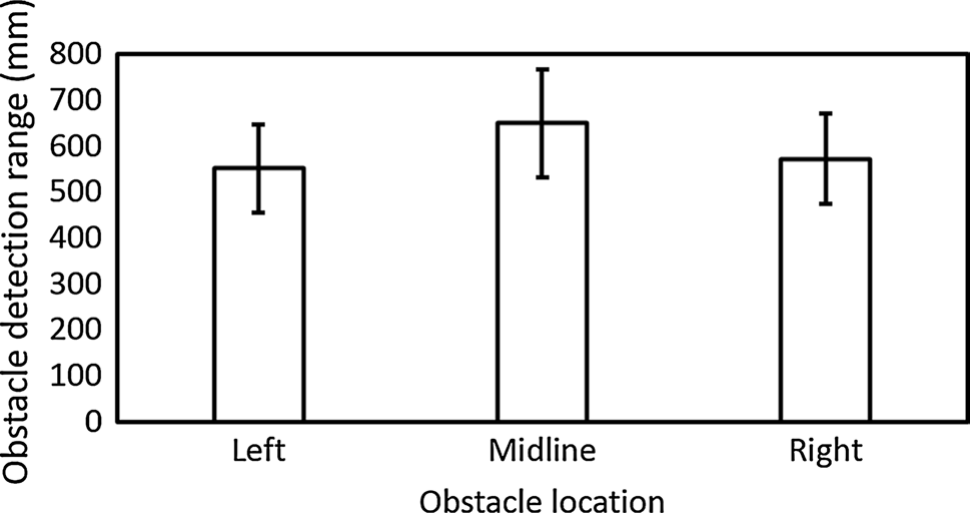
Mean obstacle detection range under auditory guidance for each obstacle location

The mean obstacle detection range using sound was 55.2, 65.1, and 57.3 cm for obstacles located to the left, midline, and right, respectively (Fig. 6). The obstacle detection range was moderately greater for obstacles located on the midline, possibly as this location provided participants with more reflected sound during obstacle approach than when the obstacle was located either leftwards or rightwards. However, there were no significant differences in obstacle detection range for the different obstacle locations [*F*(2, 18) = 0.51, *p* = 0.61] or over repetitions [*F*(2, 18) = 1.54, *p* = 0.24], and no interaction [*F*(4,36) = 0.52, *p* = 0.72].

## Discussion

The main findings are as follows: (1) under auditory guidance, sighted blindfolded participants detected the presence of an obstacle in their path on 85 % of trials and circumvented it on 67 % of the trials, suggesting that sound can be used to provide spatial layout information for locomotion when avoiding obstacles, but not always with high efficiency. (2) Buffer space was larger by a factor of 1.8 under auditory guidance than under visual guidance. (3) Movement times were greater by a factor of 27 and velocity corrections were greater by a factor of 14 under auditory guidance than under vision.

We next discuss three possible approaches to understanding how obstacle circumvention might be performed: (1) representation or model-based control, (2) information-based control, or (3) use of time-to-contact information. The representation or model-based control approach (Frenz and Lappe 2005; Turano et al. 2005) proposes that sensory information allows participants to form an internal representation of their surroundings for navigation. Under visual guidance, an individual’s surrounding space is accurately represented in relation to their action capabilities, and vision provides a constant stream of information allowing the central nervous system (CNS) to control locomotor actions in a feedforward manner (Higuchi et al. 2006). Using visual information accumulated over a series of saccades as the eye is directed to different regions of an obstacle, a comparatively rich internal representation can be built up using a process called transsaccadic integration (Prime et al. 2011). While substantial spatial layout information can be obtained using self-generated sounds (Stoffregen and Pittenger 1995; Teng et al. 2012), during locomotion internal spatial representations would need to be updated for each new self-generated sound over a relatively long time period. In contrast, transsaccadic integration under visual guidance can occur over a few hundred milliseconds, possibly resulting in more accurate internal representations during locomotion. Milne et al. (2014) suggested that head movements made while echolocating could produce sound snapshots or “echo saccades” to provide perceptual representations that are likely coarser than for vision, possibly due to lower precision using echolocation, which is poor compared to foveal acuity (Teng et al. 2012). The increased buffer space, velocity corrections, and movement times shown under auditory guidance in this study might be due to coarser internal representations of space based on sound compared to vision.

The information-based control approach (Gibson 1958; Warren 1998; Fajen and Warren 2003) proposes that the perceptual system detects information from a relevant variable (such as optic flow field variables under visual guidance) to guide movement on a moment-by-moment basis, according to some law of control (Fajen 2007). In contrast to the general-purpose perceptual cues utilized in the internal representation approach, information-based perceptual variables are task-specific. A single variable informs the individual how to perform a given task, such as negotiating the obstacle, but does not provide spatial layout information, distinguishing this approach from the internal representation approach and rendering the need for path planning or internal models unnecessary. Participants may have adapted their actions “online” (Warren 1998) to avoid the obstacle, guided by information in sound echoes during locomotion. The increased buffer space, velocity corrections, and movement times seen in our study may have been due to participants being less attuned to the acoustic information specifying passability.

A third approach involves the internal generation of “echoic tau” (Lee et al. 1992), also called “echoic time-to-contact” (Stoffregen and Pittenger 1995), to guide locomotion under auditory guidance. Echoic tau can be derived from monitoring the rate of change of an acoustic parameter such as spectral information (Stoffregen and Pittenger 1995), or the intensity difference between the emitted sound and returning echo, the echo delay, or the changing acoustic angle of the echo. This process might or might not depend on internal representations (Schiff and Oldak 1990). Lee et al. (1992) reported that echoic tau might be used by echolocating bats to govern their braking behaviour. Rosenblum et al. (2000) reported that moving blindfolded sighted echolocating participants were more accurate than stationary participants when locating the position of a removable wall, possibly due to echoic tau information being available in the moving condition.

Buffer space was larger by a factor of 1.8 under auditory guidance than when using vision. Personal space may reflect a safety margin (Graziano and Cooke 2006), and our findings suggest that while personal space can be generated using auditory information, a larger buffer space and greater caution are required than for vision. Greater buffer space under auditory guidance may also be due to increases in the variability of the walking path or postural sway while blindfolded. Franchak et al. (2012) found that under visual guidance, participants allowed larger buffers when moving through horizontal apertures compared to vertical apertures. This may be due to greater lateral sway of the body compared to vertical bounce. Paulus et al. (1984) showed that decreasing visual acuity in sighted participants using semitransparent plastic foils resulted in a proportional increase in postural sway.

Increased movement times and velocity corrections under auditory guidance are consistent with previous reports of decreased walking velocity under conditions of visual deprivation (Hallemans et al. 2010; Iosa et al. 2012; Reynard and Terrier 2015). Participants took approximately 32 s to move 1 m, equivalent to a walking speed of approximately 0.12 km/h, which is lower than velocities feasible for locomotion in daily life or that would be used by blind echolocators. Fiehler et al. (2015) suggested that sighted people new to echolocation may need to apply more conscious, high-level spatial processing to assign meaning to echoes, whereas blind people may automatically assign directional meaning to echoes for determining direction during walking. With increasing experience, the ability of sighted participants to use information from echoes may become more automatic. Consistent with this idea, over the time course of some weeks blindfolded sighted participants improve their ability to detect obstacles and avoid colliding with them (Ammons et al. 1953).

Under visual guidance, participants almost always chose the side of the obstacle that afforded the most space, despite not receiving specific instructions to do so, consistent with previous reports (Fajen and Warren 2003; Hackney et al. 2014). This was not the case under auditory guidance. Possibly the auditory information was too imprecise to allow this. However, participants may have adopted a strategy whereby they scanned the obstacle using mouth clicks until they located an edge and then moved around it, even though scanning back and forth would have allowed the participant to pass on the side affording most space. The strategy chosen for obstacle circumvention is of relevance to rehabilitation programs for those who have lost their sight; increased time spent exploring the obstacle may result in safer travel.

Using sound to generate buffer space during locomotion may be of functional importance to blind individuals, who generally detect obstacles using echolocation at similar or greater ranges than sighted individuals (Supa et al. 1944). Sensitivity to sound echoes for spatial tasks (Dufour et al. 2005; Kolarik et al. 2013) and echolocation abilities are generally increased in blind individuals (for reviews, see Stoffregen and Pittenger 1995; Kolarik et al. 2014a). Worchel and Mauney (1951) showed that training blind participants in an obstacle approach and detection task resulted in fewer collisions and greater consistency in obstacle detection and moving to its location. Increased echolocation abilities may benefit blind people when circumventing obstacles. However, this requires experimental confirmation.

The mean obstacle detection range (55–65 cm across different obstacle locations) was comparable to that measured for sighted participants (62–130 cm) detecting an obstacle using thermal noise (similar to white noise) emitted by a moving loudspeaker (Cotzin and Dallenbach 1950). Studies where sighted participants used sounds to detect an obstacle reported ranges of 1.8 m or less (Supa et al. 1944; Ammons et al. 1953). Differences in obstacle size, approach distance, experimental paradigm, training, task, room size, environment, and individual differences in abilities to use self-generated sound (Teng and Whitney 2011; Teng et al. 2012; Rowan et al. 2013) may have contributed to the different ranges across studies. The shorter approach distances we used may have resulted in the buffer space under visual guidance (19–23 cm across different obstacle locations) being smaller than for other visually guided obstacle circumvention studies: approximately 40 cm for children (Hackney et al. 2014) and approximately 60 cm for adults (Gérin-Lajoie et al. 2008).

Results from the current study and others (Supa et al. 1944; Carlson-Smith and Weiner 1996) show that auditory-guided locomotion is possible in an indoor environment, where room reverberation can sometimes benefit echolocation. Schenkman and Nilsson (2010) showed that echolocation detection performance was better in a reverberant room than in an anechoic room, possibly due to an “information surplus principle”, as changes in reverberation pattern offer a potential cue. Wallmeier and Wiegrebe (2014a) reported that for a virtual distance discrimination task using echolocation, performance was as good as or better when room reflections were present than when they were absent, similar to findings by Schörnich et al. (2012). However, long reverberation times are likely to degrade echolocation performance, as room reflections may interfere with reflections from the target object (Schörnich et al. 2012).

Gérin-Lajoie et al. (2008) suggested that a multi-sensory zone constituting personal space is used to plan safe navigation around obstacles under visual guidance. Multimodal brain areas implicated in personal space representation include the ventral intraparietal area and the polysensory zone in the precentral gyrus, whose roles may include maintenance of a safety margin around the individual and coordination of motoric actions that protect the surface of the body (Graziano and Cooke 2006). Fiehler et al. (2015) reported that echoic path direction processing was associated with brain activation in the superior parietal lobule and inferior frontal cortex in blind and sighted participants. Additional activation occurred in the inferior parietal lobule and middle and superior frontal areas in sighted echolocation novices. Kupers et al. (2010) also reported superior parietal lobule activation among blind and blindfolded sighted participants using a tactile SSD called the Tongue Display Unit in a virtual navigation task, suggesting a role of the superior parietal lobule in a navigation or route-recognition network. Occipital brain areas are involved in echoic spatial processing among blind individuals (Thaler et al. 2011; Arnott et al. 2013). Fiehler et al. (2015) highlighted that brain activation relating to sound echoes was mainly observed in parietal rather than occipital areas in their study, possibly due to task differences. Previous studies focused on spatial location only; their study focused on spatial location for locomotion.

Teng et al. (2012) reported that the spatial resolution of expert blind participants using echolocation was comparable to that observed for the visual periphery of sighted participants. In the current study, better performance under visual guidance than with audition was probably due to the better spatial acuity in the former. Visual impairment generally reduces functional spatial resolution, making visually guided obstacle circumvention more difficult. However, the effect of central or peripheral visual loss and severity of visual loss on obstacle circumvention has not been investigated and requires further study. Further investigation of auditory-guided locomotion and a visually guided condition matched in terms of acuity, for example through the use of blurring lenses, would enable a comparison of the effectiveness of visual and auditory guidance when matched in acuity.

Several studies investigated how auditory spatial perception is affected by visual loss and interpreted the results in terms of internal representations of auditory space (Lessard et al. 1998; Voss et al. 2004; Lewald 2013). Their findings suggest that representations of auditory space can be generated and maintained following severe visual loss, suggesting that audiomotor feedback is sufficient to calibrate auditory spatial representations (Lewald 2013; Lewald and Getzmann 2013). However, Wallmeier and Wiegrebe (2014a) noted that audiomotor feedback cannot conclusively explain why blind individuals demonstrate supra-normal abilities in far space (Voss et al. 2004), where feedback from self-motion cannot easily be linked to systematic changes in auditory stimuli. Echolocation provides reasonably accurate distance information (Kolarik et al. 2016), and it was recently hypothesized that echolocation may aid in calibrating auditory space (Kolarik et al. 2014a). This has been supported by Wallmeier and Wiegrebe (2014a), who showed that blind and blindfolded sighted participants discriminated distances to objects using echolocation with high acuity in far space. In terms of the internal representation approach, our findings suggest that echolocation may provide internal representations that the CNS uses to guide locomotion around an obstacle, consistent with the view that sensorymotor feedback using echolocation aids in developing an accurate spatial representation of auditory space (Vercillo et al. 2015).

In summary, the results of the current study showed that audition could be used to perform a single-obstacle circumvention task, guiding locomotion and generating buffer space in the majority of trials. However, collisions and false perceptions sometimes occurred, indicating that sound did not always provide sufficient spatial information to judge the location of an obstacle accurately.
